# Factors related to fear of movement after acute cardiac hospitalization

**DOI:** 10.1186/s12872-020-01783-9

**Published:** 2020-11-23

**Authors:** P. Keessen, C. H. M. Latour, I. C. D. van Duijvenbode, B. Visser, A. Proosdij, D. Reen, W. J. M. Scholte op Reimer

**Affiliations:** 1 Centre of Expertise Urban Vitality, Faculty of Health, Amsterdam University of Applied Sciences, Tafelbergweg 51, 1105 BD Amsterdam, The Netherlands; 2 Cardiovitaal Cardiac Rehabilitation Centre, Amsterdam, The Netherlands; 3 Department of Cardiology, Amsterdam University Medical Center, Amsterdam, The Netherlands

**Keywords:** Cardiovascular disease, Acute cardiac hospitalization, Fear of movement, Cardiac rehabilitation, Physical activity, Exercise

## Abstract

**Background:**

Fear of movement (kinesiophobia) after an acute cardiac hospitalization (ACH) is associated with reduced physical activity (PA) and non-adherence to cardiac rehabilitation (CR).

**Purpose:**

To investigate which factors are related to kinesiophobia after an ACH, and to investigate the support needs of patients in relation to PA and the uptake of CR.

**Methods:**

Patients were included 2–3 weeks after hospital discharge for ACH. The level of kinesiophobia was assessed with the Tampa Scale for Kinesiophobia (TSK-NL Heart). A score of > 28 points is defined as ‘high levels of kinesiophobia’ (HighKin) and ≤ 28 as ‘low levels of kinesiophobia’ (LowKin). Patients were invited to participate in a semi-structured interview with the fear avoidance model (FAM) as theoretical framework. Interviews continued until data-saturation was reached. All interviews were analyzed with an inductive content analysis.

**Results:**

Data-saturation was reached after 16 participants (median age 65) were included in this study after an ACH. HighKin were diagnosed in seven patients. HighKin were related to: (1) disrupted healthcare process, (2) negative beliefs and attitudes concerning PA. LowKin were related to: (1) understanding the necessity of PA, (2) experiencing social support. Patients formulated ‘tailored information and support from a health care provider’ as most important need after hospital discharge.

**Conclusion:**

This study adds to the knowledge of factors related to kinesiophobia and its influence on PA and the uptake of CR. These findings should be further validated in future studies and can be used to develop early interventions to prevent or treat kinesiophobia and stimulate the uptake of CR.

## Background

Anxiety after an acute cardiac hospitalization (ACH) is common. A recent study shows that 43% of patients suffer from anxiety at the time of ACH and 28% directly after ACH [1]. Accumulating evidence suggests that anxiety is an important risk factor for fatal and non-fatal cardiac events [2–4].

Cardiac rehabilitation (CR) is the cornerstone of secondary prevention aimed at improving physical, psychological and social functioning [5]. CR consists of multifactorial interventions such as physical activity counseling, exercise training, diet/nutritional counseling, risk factor control, patient education, psychosocial management and vocational advice [5].

Exercise training is a key element of CR and is defined as: ‘a subset of physical activity that is planned, structured, and repetitive and has as a final or an intermediate objective the improvement or maintenance of physical fitness’ [6]. Recent studies show that exercise based CR reduce cardiovascular mortality and hospital admissions and moreover improve quality of life and psychological wellbeing [7, 8]. In addition, participation in exercise based CR increases daily physical activity in patients that suffered myocardial infarction [9]. Physical activity is defined as ‘any bodily movement produced by skeletal muscles that results in energy expenditure’ [6]. During physical activity counseling, patients are encouraged to accumulate 30–60 min of moderate-intensity physical activity per day on at least 5 days of the week [9].

Despite its well know benefits, only 17% of patients perform the recommended amount of PA [10]. A potential explanation for these low PA levels, among other factors, might be anxiety experienced by patients after ACH [11]. Specifically, fear of movement (kinesiophobia) might be an important barrier to achieve adequate PA levels [12].

Kinesiophobia is described as an irrational, debilitating fear of movement and is explained by the fear avoidance model (FAM) [13]. The FAM is a biobehavioral model that describes how individuals develop chronic musculoskeletal pain (CMP) as a result of avoidance behavior based on pain related fear [13]. A recent systematic review reports that high levels of kinesiophobia (HighKin) in patients with CMP are associated with greater levels of pain intensity, disability and low quality of life [14]. In patients with coronary artery disease (CAD) HighKin are reported in 20% of the patients and are associated with decreased health-related quality of life, decreased muscle strength and reduced levels of PA [15]. In addition, patients with HighKin participate to a lesser extend in CR programs than those with low levels of kinesiophobia (LowKin) [16]. Avoidance of PA is an important predictor of major adverse cardiac events and should thus be targeted [4].

Exposure based rehabilitation programs, in which patients are gradually exposed to PA, are effective in patients with CMP [17, 18]. The setting of CR might potentially also reduce or prevent kinesiophobia after ACH by gradually exposing patients to PA [19, 20]. Participation in CR is therefore strongly recommended after ACH, especially for those with HighKin or high levels of anxiety [20, 21]. However, patients are often discharged within a short time frame which leaves little time for psychological support and patient education on the importance of CR [22].

It is unclear which factors are related to kinesiophobia, which in turn might lead to non-adherence to CR. In addition, little is known about the support needs of patients and their informal caregiver, with regards to PA and the uptake of CR. Insight in these factors might result in the development of early interventions to target or prevent kinesiophobia and to stimulate PA and the uptake of CR.

The aims of this study were therefore to explore (1) which factors are related to kinesiophobia after an ACH (2) the support needs of patients and their informal caregivers with regard to PA and the uptake of CR after ACH.

## Methods

### Design

In this qualitative study we performed semi-structured interviews with patients that were discharged after an acute cardiac event. For this study we used the COREQ checklist (Additional file 1) to assure methodological quality [23]. Recruitment of participants ended when data-saturation was reached. The Medical Ethics Committee of the Amsterdam University Medical Center approved the study protocol (protocol number: NL65218.018.18).

### Participants

Patients were enrolled in this study at hospital discharge at the Amsterdam University Medical Centre between January 2019 and July 2019. To obtain a wide variety of viewpoints, patients were included via a purposeful sampling strategy [24]. Patients were excluded if they: (a) had cognitive problems (MMSE < 24), (b) were unable to speak Dutch or, (c) were transferred to a nursing home.

### Materials

The interview guide was developed for this study and is based on the FAM which is the theoretical model for kinesiophobia [13]. The interview guide was tested in a panel, in two rounds, that individually read the interview guide and gave feedback on the first version. Afterwards the final version of the interview guide was created. The panel consisted of a patient, a physical therapist, a cardiac nurse, a psychologist and a cardiologist. The interview guide can be found in Additional file 2.

### Procedures

Patients were asked to fill in an informed consent form at hospital discharge. Patients that agreed to participate in this study were contacted by telephone 2–3 weeks after hospital discharge to arrange an interview at home or at the outpatient clinic. Prior to the start of the interview, the participant’s level of kinesiophobia was assessed with the Tampa Scale for Kinesiophobia (TSK-NL Heart). The TSK-NL Heart consists of 13 questions with a four-point answer scale with a minimum score of 13 and maximum score of 52 points. A score of > 28 is an indication of high levels of kinesiophobia [25]. This cut off score was used to divide the patients into two groups, a ‘low level of kinesiophobia’ group (LowKin) and a ‘high level of kinesiophobia’ group (HighKin). The TSK is validated in various groups of patients [26]. After the TSK-NL Heart was completed by the participant, the patient’s informal caregiver was invited to participate in the interview and to share their perspective. Each interview was recorded with a digital voice recorder. Four interviewers conducted the interviews in pairs, the first author (PK), a researcher (ICDvD), and two assistant researchers (AvP and DR), all trained by a researcher with extensive experience in conducting semi-structured interviews (CHML).

### Data analysis

For this study an inductive content analysis was used since little information about the phenomenon exists [27]. Each interview was transcribed by one of the interviewers. All interviews were assessed by PK and ICDvD. On the basis of this preliminary analysis the researchers independently assessed if new information was obtained or data saturation was reached. The transcripts were analyzed with software for qualitative data analysis (MAXQDA). A sequential coding strategy was used to analyze the transcripts. Three types of coding were used consecutively: open, axial and selective coding [27]. Initial codes were created by studying the segmented information. Afterwards, the codes were abstracted into categories and subcategories. The underlying meaning of these categories were linked together to create overall themes. All data were independently coded for categories, subcategories and themes by two researchers (PK, ICDvD). A third researcher (CHML) reviewed all codes and decided, together with PK and ICDvD, which themes were the most appropriate.

## Results

**Fig. 1:**
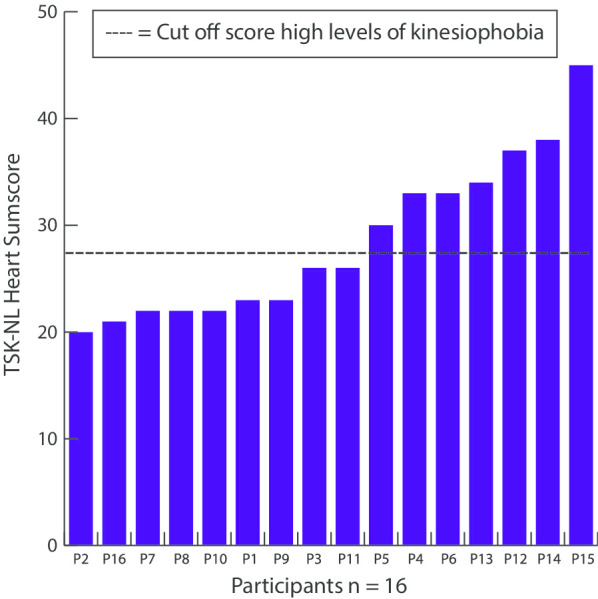
Baseline kinesiophobia scores

Data-saturation was achieved after a total of 16 patients were included in this study (Table 1), of which seven patients had high levels of kinesiophobia (Fig. 1). In six cases an informal caregiver (five spouses, one sibling) was present during the interview.

**Table 1:** Baseline characteristics

	Sex	Age range (years)	Cardiovascular diagnosis	Intervention	Cardiac disease history	Co-morbidity
*Baseline characteristics (n* = *16)*						
1	Male	60–69	NSTEMI	PCI	Stroke, hypertension	HIV
2	Female	60–69	AF	ECV	Hypertension	Lynch syndrome Colon carcinoma
3	Female	70–79	STEMI	PCI	Hypertension Hypercholesterolemia	Hypothyroid
4	Male	80–89	NSTEMI	PCI	AF	–
5	Male	70–79	AF	ECV	Stroke	–
6	Male	70–79	NSTEMI	PCI	Hypertension Hypercholesterolemia	Urothelial Carcinoma
7	Male	50–59	STEMI	PCI	Hypertension Hypercholesterolemia	
8	Male	40–49	AF	ECV	Morbus Epstein	–
9	Male	60–69	STEMI	PCI	–	–
10	Female	70–79	AF	ECV	Stroke	Hypothyroid Cholelithiasis
11	Female	50–59	STEMI	PCI	–	–
12	Male	60–69	NSTEMI	PCI	Diabetes Mellitus Hypertension	Respiratory infection OSAS
13	Female	50–59	STEMI	PCI	Hypertension Hyperglycemia	
14	Female	70–79	AHF/AF	ECV	AF Mitral insufficiency	Depression Alcohol abuse
15	Male	60–69	AHF/AF	ECV	Myocardial infarction Hypercholesterolemia Diabetes mellitus	Arthritis Lung carcinoma COPD
16	Male	40–49	AF	ECV	Myocardial infarction Hypercholesterolemia	
*TSK-NL Heart (13–52)*						
TSK-NL Heart, median (min–max)				26 (20–45)		

STEMI, ST-Elevated Myocardial Infarction; NSTEMI, Non-ST-Elevated Myocardial Infarction; PCI, Percutaneous Coronary Intervention; AF, Atrial Fibrillation; AHF, Acute Heart Failure; ECV, Electro Cardioversion; OSAS, Obstructive Sleep Apnea Syndrome; HIV, Human Immunodeficiency Virus; COPD, Chronic Obstructive Pulmonary Disease

Data were analyzed after the groups were divided into patients with HighKin and LowKin. Two themes were extracted that were related to HighKin: (1) Disrupted health care process and (2) Negative beliefs and attitude concerning physical activity. Two subsequent themes were related to LowKin: (1) Understanding necessity of physical activity after ACH, (2) Experiencing social support. One overall theme was related to support needs of patients and was defined as: Tailored information and support from health care professional.

**Fig. 2:**
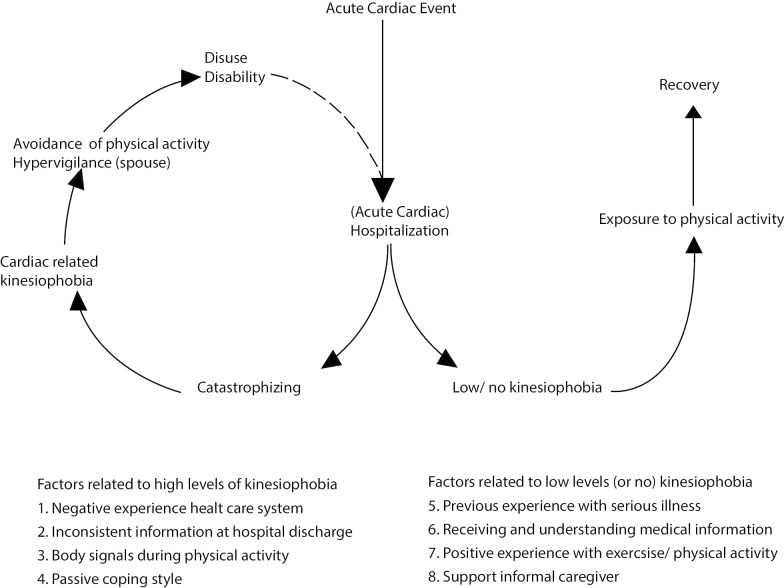
Fear avoidance model for Acute Cardiac Hospitalizations

All themes and subcategories are presented in Table 2. Based on our findings we adjusted the ‘fear avoidance model’ for patients that suffered an ACH (see Fig. 2).

**Table 2:** Identification of themes

Themes	Categories	Sub-categories
*Factors related to kinesiophobia*		
Disrupted health care process	Negative experience health care system	Reluctancy hospital
		Losing faith in the hospital
		Feeling isolated during stay
		Long waiting time for cardiac rehabilitation
		Referral problems cardiac rehabilitation
	Inconsistent information at hospital discharge	Building up Physical activity
		Cardiac event/intervention
		Side effects medication
		Impact words physician
Negative beliefs and attitudes concerning physical activity	Body signals during physical activity	Chest pain/dyspnea
		Prior experience/hypervigilance
		Side effects medication
		Serious vs innocent
		Fear of injury
		Distrusting the body
	Passive coping style	Avoidance of PA
		Preventing physical activity patient
		Hypervigilance (informal caregiver)
*Factors related to low or no kinesiophobia*		
Understanding necessity of physical activity after ACH	Previous experience serious illness	Appreciation of the value of physical activity
		Controlling co-morbidity with physical activity
		Illness spouse
	Receiving and understanding information	Health literacy
		Correct attribution body signals
	Positive experience with exercise and physical activity	Feeling healthy
Experiencing support	Social support network	Sharing stories with fellow cardiac patients
		Graded exposure to PA with informal caregiver
*Support needs*		
Tailored information and support	*Themes*	*Categories*
	Consistent information	Physical activity
		Cardiac event/intervention
		Side effects medication
	Guidance health care professional	Reassurance/trusting health care professional
		Developing an active lifestyle
		Building up physical activity
		Stimulating self-efficacy

### Factors related to high levels of kinesiophobia after an ACH

#### Theme 1: Disrupted health care process

##### Negative experience health care system

Patients with HighKin reported more negative experiences with the health care system than those LowKin. Several patients that were admitted to the cardiology ward experienced a lack of attention from the medical staff while still feeling vulnerable and anxious after their cardiac event.

I was left alone in a bed and did not see anyone during my stay at the nursing ward. There was no one that came to me to ask me how I was doing and if I was afraid. Just some human contact would make it so much better (P13).

In the weeks after hospital discharge, patients stated they missed information about their health condition, building up PA, and wanted to be in contact with a health care professional to be reassured. Several patients reported that the interval between hospital discharge and cardiac rehabilitation was too long which resulted in insecurity about their condition.

Four weeks is quite long while waiting for cardiac rehabilitation (P13).

In addition, the referral process from the hospital to CR was often unclear for patients.

The referral to CR went completely wrong. It took ages before it was clear where I needed to go and what was expected. Thinking about this makes me short of breath again (P6).

##### Inconsistent information at hospital discharge

Not receiving any, or inconsistent, information made patients feel insecure about performing and building up PA in daily life. Health care providers were often unclear in communicating about the amount of PA that the patient could do after hospital discharge.

They did not gave me any information about what I could and could not do, looking back I find this very bad (P6).

In addition, patients described that they lacked knowledge on how the body and the heart worked and what type of intervention was given to them. A lack of knowledge about building up PA and the type of intervention made patients feel insecure about performing PA as they were afraid it might lead to injury.

Someone told me that a stent can shift within the artery, if I’m not feeling well, I think about this (P13).

Furthermore, little information about side effects of medication was provided. Severe bodily reactions, caused by side effects of medication, made patients cautious about PA and in some cases led to avoidance of PA.

At hospital discharge the physician gave me a whole list with medication and then said I could go home, that’s all he said, this made me insecure (P13)

In addition to clear and consistent information, patients also wanted to be reassured by their physician before leaving the hospital.

I really missed talking to my physician about what had happened to my heart before I left the hospital (P14).

Physician’s words were found to influence patients’ beliefs about their physical state and thereby also the amount of PA they could perform in daily life.

The doctor told me to keep calm, keep calm, keep calm, this just goes through my head the whole time (P12).

#### Theme 2: Negative beliefs and attitude concerning physical activity

##### Distressing body signals during physical activity

Body signals such as: chest pain, dyspnea, pain in the jaw and extreme fatigue, were often experienced as distressing and made patients fearful about PA.

In some cases, these body signals were caused by side effects of medication.

I felt a weird pressure on my chest, like my heart skipped a beat. I panicked, so I went back to the emergency room where they examined me. Afterwards they told me it was a side effect of metropolol (P13).

Experiencing severe bodily signals made patients alert and in some cases hypervigilant.

If my heart skips a beat, I don’t do anything, I’m really anxious to become short of breath (P15)

It was difficult for patients to discriminate between serious and innocent body signals. Patients described that they often felt like they were experiencing a new cardiac event and did not know how to interpret their body signals.

I don’t have to do anything, if I just breath in a certain way it feels like it can go wrong again (P14).

Sometimes I feel a slight sting in my chest and I don’t know what to do (P13).

Participants stated that they were afraid that PA could harm their body. As a consequence, these participants often distrusted their body and performed little PA.

I don’t know if my heart is damaged and what I can and cannot do (P6)

##### Passive coping style

Due to the impact of the event, several patients developed a passive coping style and avoided PA.

I did not do anything for six weeks, I’m just staying in bed and on the couch, I can’t do much more (P12).

P14 stated that she did want to do any PA, even when her partner asked her to join him.

Daily, my husband asks me if I will join him for a walk, every day I decline,

I know physical activity is healthy but I just don’t do it, I was never a sporty type (P14).

In addition, this P14 stated that participating in CR did not work for her.

Talking about developing an active lifestyle, three years ago I participated in CR and I talked to a psychologist, but these tricks do not work for me (P14).

Several informal caregivers became hypervigilant which resulted in withholding patients from any household tasks or other forms of PA.

If I do too much and I get complaints, my husband becomes angry and tells me to sit down. (P13).

My husband does all the groceries and cooking and tells me to relax (P14).

Patients with HighKin often avoided PA and developed a passive coping style. In several cases, this maladaptive coping style was facilitated by the informal caregiver as a result of hypervigilance.

## Additional results

Although, two patients, P3 and P11 respectfully, were classified as patients with LowKin their quotes expressed signs of HighKin.

### Negative experience health care system

P11 reported that the hospital was reluctant with admitting her to the emergency room as it was suspected that her complaints were non-specific. After the general practitioner insisted an immediate admission to the cardiac care unit, she was diagnosed with a NSTEMI.

The interventional cardiologist said afterwards that it was good I listened to my body (P11).

Due to this incident, the patient stated she lost trust in her body and eventually became hypervigilant about body signals.

### Inconsistent information at hospital discharge

In addition P11 reported to miss information which made her insecure about suffering another cardiac event.

They didn’t tell me anything at the hospital and it passed by so quick. Who says I won’t suffer another myocardial infarction? (P11).

P11 also reported that she suffered from side effects of the statins which impacted her ability to move.

I missed information about medication and side effects’. At first, I felt better than after my myocardial infarction, until the statins began to work. I sat on the couch like a dead bird, muscle ache everywhere and unable to move (P11).

P3 reported that she did not know if physical activity would injure her body.

I don’t know what I can and cannot do and if I injure my body if I do any physical activity (P3).

What is my heart able to handle? Not knowing this, is very annoying (P3).

P3 also reported that the information about PA, provided at the hospital, was unclear.

What does it mean to take it easy? The doctor told me I could do the same amount of physical activity as before the event, but it’s still unclear how much I can do, I should have asked (P3).

### Distressing body signals during physical activity

P11 stated that experiencing body signals made her anxious.

And when I am sitting on the couch, or walking around, I feel every sting. That frightens me. (P11).

In addition, P11 also stated that it was difficult to discriminate between body signals.

How can I determine the severity of my body signals? (P3).

I don’t know what I can do and cannot do and if I injure my body if I do any physical activity, my heart suffered a big blow and even though I do not feel this every second, I do know it, you never know what may happen next. (P3).

### Factors related to low levels of kinesiophobvia after an ACH

#### Theme 1: Understanding necessity of physical activity after ACH

##### Previous experience with recurrent (serious) illness

Patients with LowKin often stated that they were not afraid to move due to previous hospital admissions or co-morbidity.

I’m not afraid to move, I just can’t keep my balance when I’m walking due to the stroke I have suffered. Before my stroke I was very active, but now I’m just so tired (P10).

I’ve had more than twenty cardioversions already, I know how it works, I won’t let it rule my life. (P8).

Some patients reported to have comorbidities that required physical activity to control it.

I have to do exercise in order to control my diabetes. (P9).

Prior experience with being admitted to the hospital was related to LowKin. In addition, having a co-morbidity that required exercise, motivated patients to be physically active or perform exercise, which in turn might have prevented the occurrence of kinesiophobia.

##### Receiving and understanding medical information

Patients with LowKin reported that they received consistent information from their physician and felt that the physician took the time to answer their questions which made them feel reassured.

I was really relieved that he said there was no acute danger and that I could do whatever I wanted, he really asked me a lot of things and I felt he really listened to me (P2).

Side effects of medication often caused unpleasant body signals. Patients with LowKin were more inclined to attribute unpleasant body signals to side effects of medication than to a new cardiac event.

The prescription said that these pills could cause pain under the sternum, so whenever I feel a pain in my chest, I say it’s due to a side effect of my medication (P2)

Giving time to patients to ask questions and providing clear and consistent information made patients feel more confident about performing PA. Besides talking to a physician, these patients also felt reassured by reading information about side effects of their medication. Being able to read and understand medical information and correctly interpreting body signals related to side effects might lead to LowKin.

##### Positive experience with exercise

Most patients with LowKin had a positive experience with exercise and were thus more inclined to do exercise despite their ACH.

I always feel better when I return home after doing exercise (P2).

When I am playing volleyball I completely forget everything else (P8).

Some patients already participated in CR and were looking forward to participating again since CR was a positive experience for them.

After my previous cardiac event I immediately wanted to start training just to feel better, I can’t wait for it. (P7).

#### Theme 2: Experiencing support

##### Social support

Patients with LowKin felt supported by talking to people that went through comparable experiences.

It’s not just about the rehabilitation but it’s also about drinking coffee together and sharing experiences, that also helps me (P9).

These patients also felt supported by their informal caregiver that helped them building up PA levels.

Last weekend I went for a walk with my neighbor. I was a bit anxious so we walked for a short while and that felt good. She really helped me through (P2).

### Support needs in patients and spouses with regards to kinesiophobia and participation in CR

Patients, both with HighKin and LowKin, and spouses were asked what they needed in order to increase PA levels and participate in CR after an ACH.

#### Theme: Tailored information and support from health care professional

##### Receiving consistent information

Patients stated that they wanted more detailed information about PA during hospital discharge.

I just want some simple information about what I can and cannot do, can I walk the stairs? Can I drive my car? (P11)

Patients also wanted to learn more about the side effects of certain types of medication since this caused distressing body signals which in turn led to avoidance of PA.

I would like to know why I have to take those pills, I had lots of side effects. (P12)

Furthermore, patients wanted more background information about their cardiac event and the intervention they received.

It’s not a small thing, having a heart attack. In the hospital you don’t know what’s going on and when you leave you still don’t know (P11)

For several patients it was unclear what to expect from CR. They had many questions about the aim of CR and did not know what to expect.

What is there to rehabilitate about the heart? (P3).

##### Guidance health care professional

Patients wanted to be reassured by a physician, before they started increasing their PA levels, as they often felt insecure about their heart. Some patients said they would be more confident if they would be monitored continuously.

It would be great if there would be someone next to you all the time to make an ECG and tell you nothing is wrong (P3).

In addition, patients said they wanted to be reassured about their physical state by talking to their physician.

Certain things I would like to have re-confirmed (P14)

Trust in health care professionals was noted as an important prerequisite to perform more PA.

Trusting caregivers, cardiologist, nurse practitioners, physiotherapists is really important (P3)

Patients stated that they want to be confident enough to perform daily PA.

I want be confident again that I don’t injure myself, by walking stairs or walking for miles (P3).

It was also reported by patients that they wanted to be able to gain confidence and do exercise by themselves.

I want to participate in CR to gain confidence so that afterwards I can start exercising alone (P7)

In the period before cardiac rehabilitation, patients stated that they felt anxious about doing PA without the guidance from a physical therapist.

I would feel anxious if I started exercising without guidance. It’s about confidence. I can do it, but it would not feel right (P7).

Patients reported that they hoped cardiac rehabilitation would help them build up a more active lifestyle.

I hope that I will benefit from cardiac rehabilitation and that afterwards I will be able to take the bike instead of the car to do my groceries (P6)

One patient chose to build up PA by herself and stated that she wanted more information about the type of exercises that were recommended.

Should I do interval training or focus more on strength training (P2)

## Discussion

This study shows that HighKin are related to a disrupted health care process and negative beliefs and attitude concerning physical activity. LowKin after an ACH are related to understanding the necessity of physical activity and experiencing support. Patients in both groups (HighKin and LowKin) stated that they needed tailored information and support from a health care provider after hospital discharge.

Some patients and informal caregivers reported a negative experience with the health care system after their hospital admission was denied due to hospital crowding. In some cases, this resulted in significant stress in patients and informal caregivers. Previous studies have found that hospital crowding is related to lower patient satisfaction [28], patients not receiving the appropriate care [29] and complications during the hospital stay [30] which in turn can lead to HighKin [15]. This finding emphasizes the need for greater attention to the negative psychological effects of hospital crowding on patients and informal caregivers. Other aspects related to HighKin are a long waiting time until CR or problems with referral to CR. Although current guidelines state that CR should start 28 days after the referral (42 days for CABG) [20], patients reported that time until the start of CR was too long which made them feel insecure about being physically active. Feeling anxious after a cardiac event is associated with delays in seeking medical help and the adoption of an unhealthy lifestyle [31] and might thus lead to delayed participation in CR which negatively impacts fitness outcomes of patients [32]. A recent study shows that an active lifestyle (self-reported) at the hospital ward, after an ACS, is associated with reduced risk of new cardiac events. An early intervention (e.g. before discharge from hospital) that stimulates PA might potentially prevent kinesiophobia and the recurrence of cardiac events [33].

Experiencing a lack of consistent information at hospital discharge contributed HighKin since patients lacked knowledge about building up PA levels after an ACH. Not receiving information, especially about the safety of PA, is a well-known barrier for PA in patients with cardiovascular disease [34]. When information is provided, it might lack clarity due to vague or inconsistent language. An example of vague information with respect to PA is the advice to `take it easy’ or to `just do the same amount of PA as before the event’. This is an important finding since research shows that health care providers’ orientations towards illness predicts perceived harmfulness of PA in patients [35]. Therefore, health care providers must be as clear and consistent as possible when providing information on PA.

Patients often had difficulties in discriminating between `harmful’ and `harmless’ body signals, which were often caused by stress or side effects of medication. Before hospital discharge, patients should be educated on how to discriminate between body signals. Not being able to do this, might result in avoidance of PA and unnecessary hospital visits. Many patients in this study believed that PA could result in injury to their heart. In our previous study, we validated the TSK-NL Heart questionnaire, and showed that `fear of injury’ is the main factor in the construct `kinesiophobia’ [25]. Fear of injury after an acute cardiac event is normal and understandable. However, excessive fear of injury results in ineffective coping strategies, such as avoidance of PA, which might lead to further disability and possible secondary cardiac events [24]. Patients with little understanding of pain mechanisms tend to perceive their body signals as more threatening or dangerous due to fear of injury, eventually leading to more catastrophic thoughts and less adaptive coping strategies [36]. Insight in the origin of body signals and learning effective coping strategies might prevent avoidance behavior in patients after an ACH.

In several cases, informal caregivers performed all physical tasks (household, groceries) as a result of hypervigilance. Research suggest that hypervigilance in spouses after a cardiac event, although well intended, may undermine the patient’s health and recovery [37] and should thus be targeted.

Several patients reported to have LowKin due to prior experience with (severe) illness. This finding is line with the study of Bäck et al. (2013), which suggested that suppression of previous experience of heart failure was related to LowKin [15]. In this study, some patients had experience with controlling their comorbidity (e.g. diabetes) by doing exercise and were therefore motivated to be physically active, or perform exercise, which in turn might have led to lower levels of kinesiophobia. Patients with LowKin stated, in contrast to those with HighKin, that they received consistent information and also felt they were given the opportunity to ask questions before leaving the hospital. Moreover, these patients attributed their body signals to side effects of medication and felt reassured by carefully reading the medication prescription instead of attributing their body signals to a new cardiac event. A previously conducted systematic review found that poor ability to obtain and understand medical information (health literacy) is consistently associated with anxiety, readmissions and lower social support [38]. This finding might also apply to patients with HighKin and emphasizes the need for accessible and understandable information for (all) patients after an ACH.

Patients that had experience with exercise, or already participated in CR, were more positive about PA and exercise and were looking forward to starting CR. Previous experience with exercise has been reported by Bäck et al. (2017) as an important facilitator of participation in CR [39]. Furthermore, these patients felt supported by patients that went through the same experience during CR. Talking about their experiences relieved their anxiety and kinesiophobia. These patients also felt more support from their informal caregivers who gradually exposed them to greater levels of PA. Graded exposure to PA is an effective method to overcome kinesiophobia in patients with low back pain [15] and might also be suitable for patients with cardiovascular disease.

Patients stated that they wanted consistent information about their cardiac event and cardiac intervention, body signals, side effects of medication and health benefits of CR before hospital discharge. Aside from consistent information, patients wanted to be reassured by a health care professional when building up PA in their daily lives. Being well informed and reassured, in the early phase after an ACH, is vital since 1 in 5 patients drop out of CR as a result of feeling anxious [40]. Targeting anxiety and kinesiophobia in the early phase after an ACH before the start of (physical) CR might alter participation rates in CR [39].

### Strengths and limitations

This study has several strengths. To our knowledge this is the first study that explores kinesiophobia after an ACH with an inductive content analysis. A variety of patients with different ages, gender, diagnoses and co-morbidities were included in this study. We consider this a strength since we wanted to explore viewpoints from a variety of participants that were eligible for CR.

This study also has limitations. Firstly, we used semi-structured interviews to obtain our data. During semi-structured interviews, the interviewer actively participates which in turn might lead to bias due to personal prejudices about the topic. Secondly, content analysis is subject to error especially due to interpretation of the data. To minimize the chance of misinterpretation and to increase the trustworthiness of the results, triangulation was used. Thirdly, we aimed to explore the concept of kinesiophobia in a heterogeneous cohort using a purposeful sampling strategy which in turn impacts the generalizability of the results. Nevertheless, due to the qualitative research design, meaningful, in-depth data was obtained concerning experiences, beliefs and barriers in patients with varying backgrounds. Lastly, we used the TSK-NL Heart to define the level of kinesiophobia in cardiac patients were the cut-off score of > 28 points is used to define high levels of kinesiophobia [9]. A single cut-off point might be suitable for practical reasons like admission to an intervention but the concept of fear in itself is not dichotomous. Our data suggest that the cut-off point of 28 might be too high. Two patients (P11, P3) in our study scored slightly under the cutoff with a score of 26 points. Based on their interviews they seemed to be misclassified since they reported several signs of kinesiophobia. A recent study suggests using categories to classify the severity of kinesiophobia as follows: subclinical: 13–22; mild: 23–32; moderate: 33–42; and severe: 43–52 [22]. According to this classification, several patients in our study population would have been identified with mild kinesiophobia. It’s unknown if the above mentioned categories are applicable for patients with cardiovascular disease. More research is needed to assess the validity of these cut off points for the TSK-NL Heart.

This study reveals factors related to kinesiophobia after an ACH and the support needs of patients and informal caregivers. These findings need be further investigated in studies with a quantitative research design and can be used to develop early interventions to target or prevent kinesiophobia after an ACH.

## Conclusion

The findings of this exploratory study suggest that `a disrupted health care process’ and `negative beliefs and attitudes concerning physical activity’ are related to high levels of kinesiophobia after an ACH. On the other hand, understanding the necessity of PA and experiencing social support after ACH, are related to low levels of kinesiophobia. Patients reported `tailored information and support from a health care professional’ as most important needs after ACH. These findings can be used to make health care professionals more aware of patients’ needs after ACH and thereby taking into account the possible role of kinesiophobia in the health care process. The results of this study need to be further investigated in studies with a quantitative study design and can be used to develop early intervention strategies to prevent kinesiophobia, stimulate physical activity and the uptake of CR.

## Supplementary information

**Additional file 1**. COREQ checklist.

**Additional file 2**. Interveiw guide.

## Data Availability

The dataset and interview guide, used during the current study, are available from the corresponding author on reasonable request.

## Abbreviations

CAD: Coronary Artery Disease
ACH: Acute Cardiac Hospitalization
PA: Physical Activity
CR: Cardiac Rehabilitation
STEMI: ST-Elevated Myocardial Infarction
NSTEMI: Non ST-Elevated Myocardial Infarction
AF: Atrial Fibrillation
AHF: Acute Heart Failure
ECV: Electro CardioVersion
PCI: Percutaneous Coronary Intervention
HighKin: High levels of Kineseiophobia
LowKin: Low levels of Kinesiophobia
