# Supplementary material for: Factors related to fear of movement after acute cardiac hospitalization
Source: BMC Cardiovasc Disord. 2020 Nov 23;20:495. doi: 10.1186/s12872-020-01783-9 (PMC7686769; doi:10.1186/s12872-020-01783-9)
Supplement: Supplementary file 1 — Additional file 1. COREQ checklist. [file 12872_2020_1783_MOESM1_ESM.docx]

Additional file 1: Appendix 1

| **Main topics** | **Example of follow-up questions** |
| --- | --- |
| Patient’s and informal caregivers experiences during cardiac event | - Can you tell us about what happened during your cardiac event? |
| Experiences during hospitalization | - What was your experience at the hospital? - How was it to do physical activity at the hospital after the event? - Did you receive any help or instructions from a health care professional (cardiologist/registered nurse/physiotherapist)? - Can you tell us about the discharge process? |
| Experiences after hospital discharge | - What are your experiences after hospital discharge - How was it to do daily physical activities directly after hospital discharge? - Did you receive support or do you receive support now? - Where there any barriers for physical activity after hospital discharge and are there any barriers now? - What are your beliefs about physical activity? - Do you avoid certain types of physical activity? - If you look back at your experience, what would you have liked to see different? (information/guidance/treatment). |
| Cardiac rehabilitation | - Are you planning to participate in cardiac rehabilitation? |
